# Carbon gain in upper but loss in deeper cropland soils across China over the last four decades

**DOI:** 10.1073/pnas.2422371122

**Published:** 2024-12-31

**Authors:** Zhenghu Zhou, Chuankuan Wang, Yu’e Li, Xuhui Wang, Xinhua He, Minggang Xu, Andong Cai

**Affiliations:** ^a^School of Ecology and Northeast Asia Biodiversity Research Center, Northeast Forestry University, Harbin 150040, China; ^b^Institute of Environment and Sustainable Development in Agriculture, Chinese Academy of Agricultural Sciences, Beijing 100081, China; ^c^Institute of Carbon Neutrality, Sino-French Institute for Earth System Science, College of Urban and Environmental Sciences, Peking University, Beijing 100871, China; ^d^Institute of Eco-environment and Industrial Technology, Shanxi Agricultural University, Taiyuan 030031, China; ^e^School of Biological Sciences, University of Western Australia, Perth, WA 6009, Australia; ^f^Department of Land, Air and Water Resources, University of California at Davis, Davis, CA 95616; ^g^State Key Laboratory of Efficient Utilization of Arid and Semi-arid Arable Land in Northern China, Institute of Agricultural Resources and Regional Planning, Chinese Academy of Agricultural Sciences, Beijing 100081, China

**Keywords:** soil organic carbon, soil profile, carbon inputs, climate change, resampling

## Abstract

Increasing soil organic carbon (SOC) in agricultural systems is a primary nature-based option for mitigating climate change, improving soil fertility, and ensuring food security. However, the consequences of global warming and increases in carbon inputs on cropland SOC stocks over the last few decades remain largely unknown, particularly in deeper soil layers. Here, by using repeated measurements, we reassess variations in SOC stocks across a 0 to 100 cm soil profile at the same locations in China’s upland croplands in 1980 and 2023. We observe an overall net accumulation of 0.74 Pg SOC (7%) with a mean sequestration rate of 13.7 g SOC m^−2^ y^−1^. This accumulation results from 0.86 Pg SOC gains at upper soil depths (0 to 60 cm) induced by carbon inputs, alongside 0.12 Pg SOC losses at deeper soil layers (60 to 100 cm) prompted by warming-enhanced decomposition. While our findings indicate a successful, though lower than expected, increase in overall net SOC stocks, the loss of more recalcitrant deeper SOC warrants further consideration of its effects on enhancing cropland SOC accumulation to achieve carbon neutrality over the long term.

## Significance

Reports of soil organic carbon (SOC) dynamics in China’s croplands vary substantially due to diverse data sources and the lack of a standardized methodology. Additionally, SOC dynamics in deeper soil often receive insufficient attention. By conducting uniformly methodological resampling of whole-soil profiles at the same locations in 1980 and 2023, we present a national atla of SOC dynamic. Significantly, an overall net accumulation of 0.74 Pg SOC results from gains of 0.86 Pg SOC at the upper 0 to 60 cm soil layers but losses of 0.12 Pg SOC at the deeper 60 to 100 cm soil layers due to intensified decomposition driven by increased temperatures. Our findings provide insights into SOC responses to climate warming and agricultural carbon accumulation strategies.

Soils are the largest carbon pool in terrestrial ecosystems, storing three times more carbon than atmosphere and two times more carbon than vegetation (1, 2). Hundreds of manipulation experiments have investigated the impacts of climate warming on soil organic carbon (SOC) dynamics (3). However, there is no consensus on the magnitude of SOC feedback to climate warming, which is partly due to the brief duration of most experiments compared with the lengthy turnover of SOC, as well as the distinction between experimental warming and ambient ecosystem warming in reality (3, 4). Large-scale soil resampling studies across broad geoclimatic gradients can deliver reliable estimates of regional-scale SOC changes over time, which reflect the responses of SOC to warming (5, 6). In addition, the scarcity of observation-based quantification of global temporal variations of SOC, as a benchmark, also constrains the precision of Earth System Models in predicting the effects of climate change on SOC cycling (4, 5).

Soils are estimated to contribute up to 25% of the potential of natural climate solutions for climate change mitigation, particularly in agricultural croplands (2). Aside from the mitigation of climate change, increases in SOC in agricultural ecosystems also enhance soil quality and food security (1). The sequestration of SOC in agricultural soil is a hot topic in major policy initiatives (1) and carbon credit issues (7). For example, upland croplands are hotspots of proposed natural climate solutions like the 4 per mille initiative (1, 8). In previous studies, economics- and policy-driven increases in carbon inputs to soil, such as continuous variety regeneration and increases in irrigation, fertilization, and grain yield (*SI Appendix*, Fig. S1), were found to improve SOC accumulation in the 0 to 20 cm topsoil of Chinese croplands (9, 10). However, the responses of deeper SOC to global change differ from surface SOC because the abiotic and biotic properties regulating SOC cycling vary with soil depth (11). In the long term, the slow turnover of deeper SOC is critical for terrestrial feedback to climate change (11). However, it is largely unknown whether deeper SOC shows the same temporal pattern as topsoil in cropland.

**Fig. 1.**
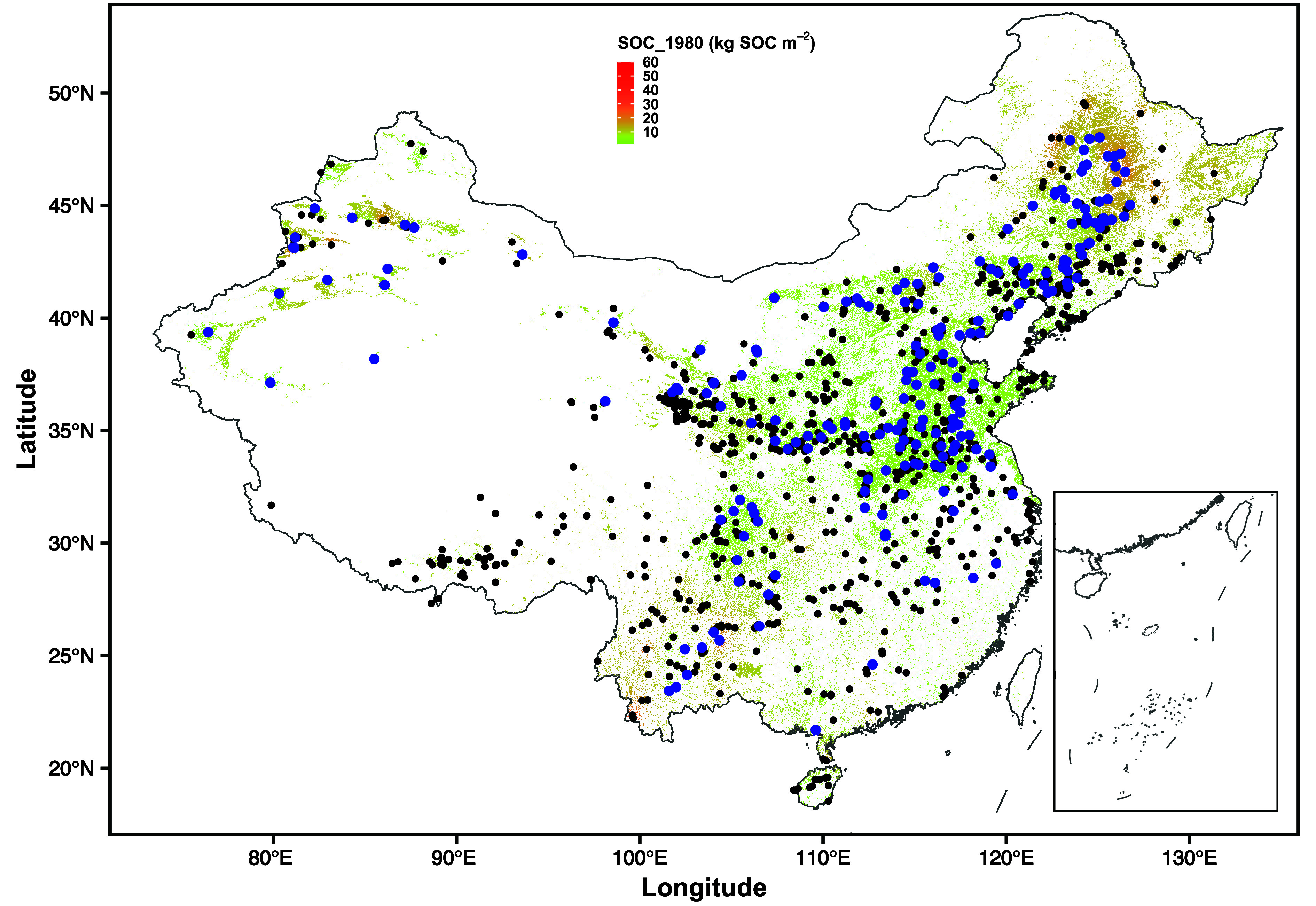
Spatial distribution of 205 resampling sites across China’s upland croplands. Blue and black dots represent sample sites in 2023 and 1980, respectively. The depth of resampling is 100 cm (0 to 20, 20 to 40, 40 to 60, and 60 to 100 cm). Shaded areas are soil organic carbon (SOC) stocks in 1980 (SOC_1980).

Here, we conducted a large-scale resampling survey to reveal the spatial patterns of SOC stock changes encompassing an entire one-meter soil profile (0 to 20, 20 to 40, 40 to 60, and 60 to 100 cm) across China’s upland croplands. This survey leveraged datasets mainly from 1980 (the Second National Soil Survey) and a subset of resampled sites from 2023. The 2023 subset included 205 selected sites that were resampled from the original 1980 sampling sites (site by site), spanning approximately 25° latitude and 50° longitude (Fig. 1). We employed the following factors to investigate drivers of the magnitude of SOC storage changes: climate factors (mean annual temperature, MAT; mean annual precipitation, MAP; MAT and MAP changes over the last four decades; extreme high-temperature and drought events; *SI Appendix*, Fig. S2), management practices (changes in fertilization, grain yields, and straw return), original SOC stocks in 1980, net primary productivity, and soil properties (pH and texture). Based on these datasets, we explored the temporal dynamics of SOC across entire one-meter soil profiles over the last four decades.

## Results and Discussion

**Fig. 2.**
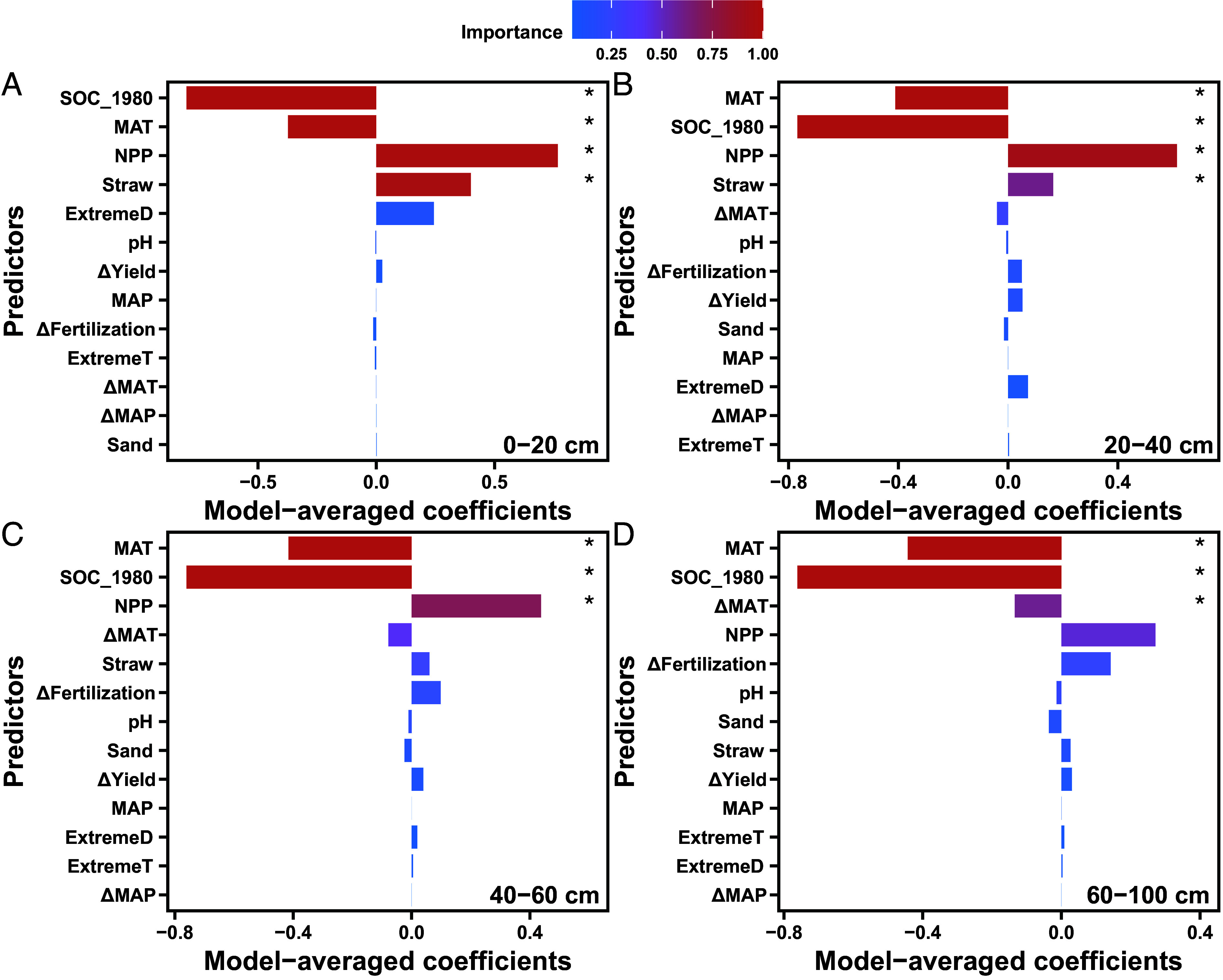
Factors that influenced changes in SOC stocks for soil depths of 0 to 20 (*A*), 20 to 40 (*B*), 40 to 60 (*C*), and 60 to 100 cm (*D*). Asterisks indicate factors that were included by the best model (*SI Appendix*, Table S1). SOC_1980, SOC stock in 1980 (natural logarithm-transformed). NPP, net primary productivity. MAT, mean annual temperature. MAP, mean annual precipitation. ΔMAT, changes in MAT in the past four decades. ΔMAP, changes in MAP over the last four decades. ΔFertilization, changes in fertilization over the last four decades. ΔYield, changes in grain yields over the last four decades. Straw, proportion of straw return. ExtremeT, frequency of extreme high-temperature events. ExtremeD, frequency of extreme drought events.

Our study quantifies SOC stock changes across an entire one-meter soil profile in China’s croplands. Our site-by-site resampling revealed significant variations in SOC stocks across the 205 sites and soil depths (*SI Appendix*, Fig. S3). Model selection analysis identified the original 1980 SOC stocks and MAT as the two primary drivers of SOC stock changes across all four soil depths (Fig. 2 and *SI Appendix*, Table S1 and Fig. S4). The negative effect of the original SOC stocks on SOC stock changes was consistent with findings from England and Wales (6) and Europe (12). However, the threshold at which soil acted as a carbon source versus a sink decreased with soil depth, with 3.4, 1.9, 1.5, and 2.6 kg SOC m^−2^ at the 0 to 20, 20 to 40, 40 to 60, and 60 to 100 cm soil depths, respectively (*SI Appendix*, Fig. S5). Consequently, the subsoil had a weaker capacity to hold the original high SOC than the topsoil did over the last four decades.

High MAT was expected to negatively impact SOC stock changes, as increased temperatures typically accelerate SOC loss by accelerating decomposition. The importance of net primary productivity and straw return in regulating SOC stock changes decreased with soil depth, while the negative impacts of MAT changes on SOC stock changes were identified by the best model for the deeper 60 to 100 cm depth (Fig. 2 and *SI Appendix*, Table S1 and Fig. S4). The loss of deeper SOC could therefore be exacerbated by climate warming. The following four potential mechanisms may explain the warming-induced loss of SOC in deeper soils. First, a whole-soil-profile warming experiment at the University of California’s Blodgett Experimental Forest revealed that warming resulted in less SOC in the warmed subsoil relative to the control, but more SOC in the topsoil (13, 14). Second, the decomposition of organic matter is more sensitive to temperature in subsoil than in topsoil (15). Third, the plow hardpan in croplands prevents the transfer of crop residues from the topsoil to subsoil (16). Fourth, the continued expansion of irrigated farmland and climate change-induced increases in precipitation in China (*SI Appendix*, Figs. S1 and S2) have resulted in shallower rooting profiles due to increases in water availability, leading to lower carbon inputs into deeper soils (17, 18). Although extreme high-temperature and drought events were not accepted by the best models (Fig. 2 and *SI Appendix*, Table S1), we found significantly negative effects of extreme climate events on SOC sequestration at the 0 to 20 cm topsoil of the Chinese croplands (*SI Appendix*, Fig. S6). Changes in climate extremes can influence long-term SOC dynamics by altering soil physicochemical properties, plant carbon inputs, microbial physiology, and decomposition rates (19). Here, our results demonstrate that climate extremes play a key role in determining SOC balance over the long timescale.

**Fig. 3.**
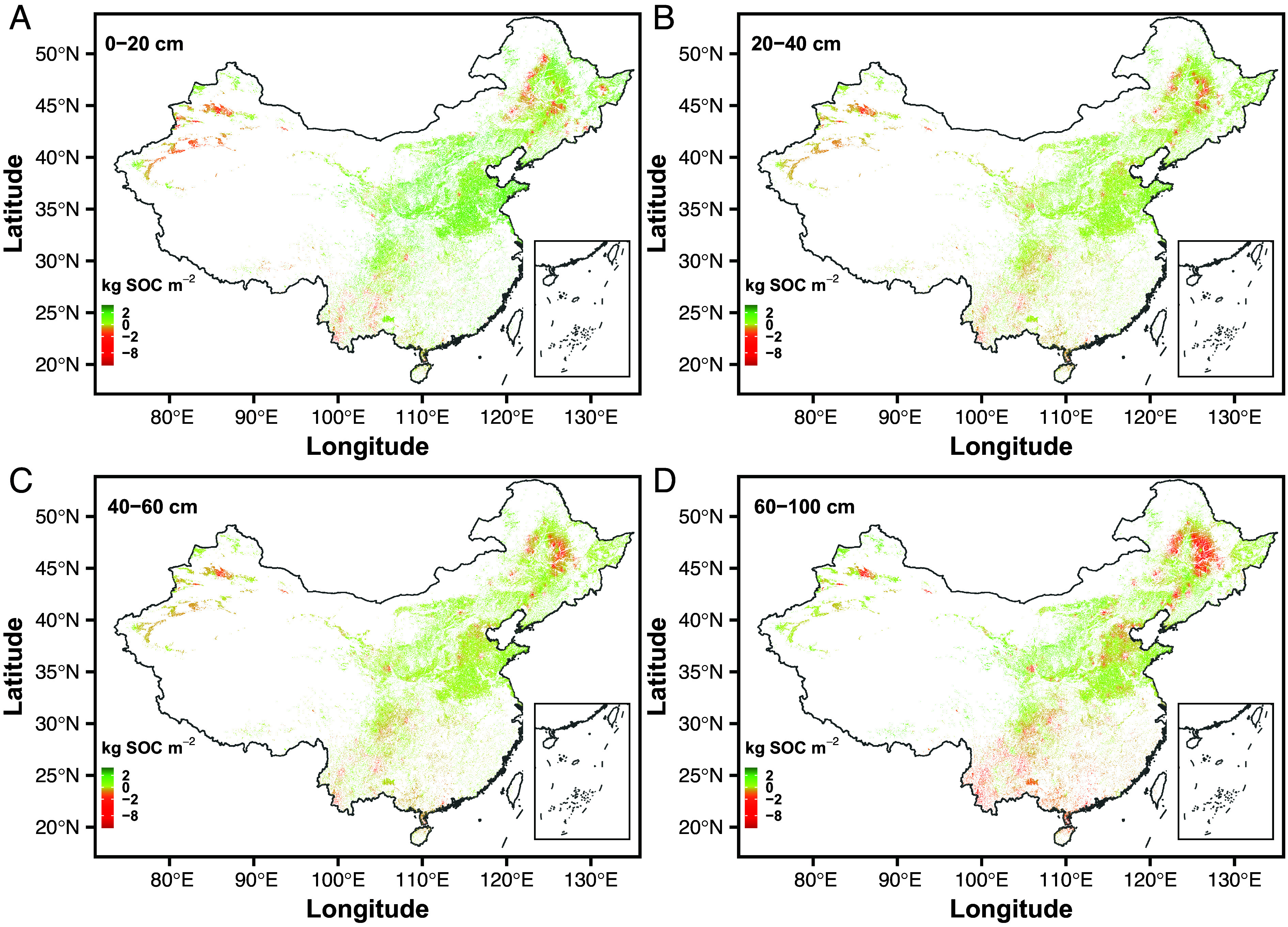
Spatial distributions of SOC stock changes by soil depths of 0 to 20 (*A*), 20 to 40 (*B*), 40 to 60 (*C*), and 60 to 100 cm (*D*).

**Fig. 4.**
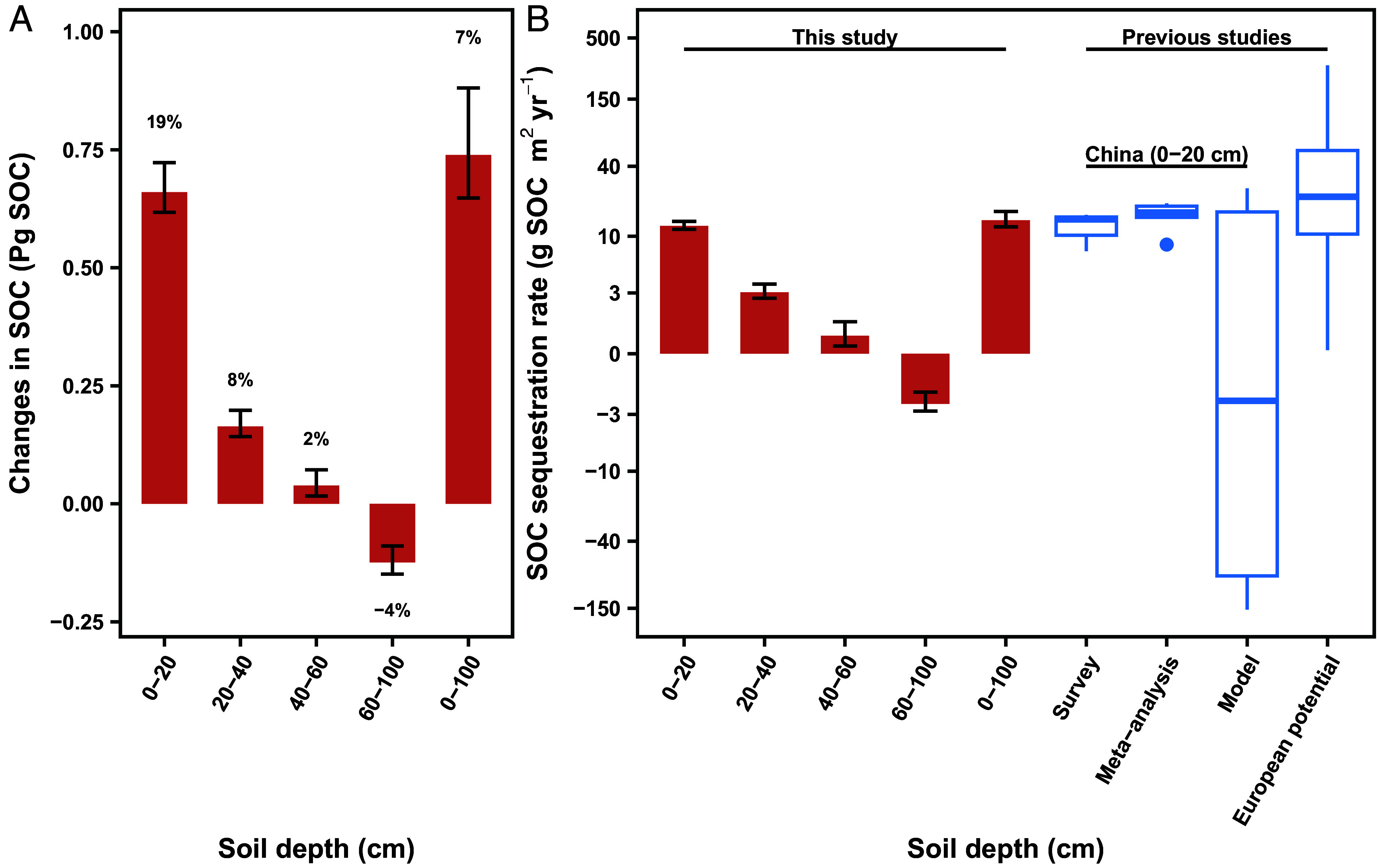
Changes in SOC stocks in Chinese croplands (*A*) and previous studies (*B*). Error bars are 5% and 95% percentiles from 100-times bootstrapping. In the boxplot, the center line is the median, box limits are upper and lower quartiles, and whiskers are 1.5 times the interquartile range. Individual data points from previous studies are shown in *SI Appendix*, Table S2.

Using the best models, we estimated SOC balances on a national scale by soil depths (Fig. 3). Significant SOC gains were found at depths of 0 to 20 cm ($0.66_{0.62}^{0.72}$ Pg SOC, *t* test *P* < 0.001) (mean with 5% and 95% percentiles), 20 to 40 cm ($0.16_{0.14}^{0.20}$ Pg SOC, *t* test *P* < 0.001), and 40 to 60 cm ($0.04_{0.02}^{0.07}$ Pg SOC, *t* test *P* < 0.001). We found a significant SOC loss at 60 to 100 cm depth (${-0.12}_{-0.15}^{-0.09}$ Pg SOC, *t* test *P* < 0.001) (Figs. 3 and 4). Overall, we observe an overall net accumulation of 0.74 Pg SOC (7%) across a 0 to 100 cm soil profile. At 0 to 20 cm depth, the SOC sequestration rate was $12.3_{11.5}^{13.4}$ g SOC m^−2^ yr^−1^, closely aligning with rates reported in previous surveys (9, 20, 21) and meta-analyses (22–27), albeit it was greater than that from the topsoil prediction models for China (28–33) (Fig. 4 and *SI Appendix*, Table S2). The observed SOC sequestration rates in Chinese croplands were also compared with potential future rates in Europe (Fig. 4) using recent country-specific estimates (34).

However, focusing only on topsoil cannot fully elucidate the general responses of soils to climate change, as deeper SOC dynamics comprise one of the largest domains of uncertainty in global carbon balances (11). In this study, we observed a significant loss of SOC at the 60 to 100 cm depth, which highlights the challenges of SOC management at deeper soil depths. Sustainable cropland management, including no-till and straw return, resulted in a topsoil carbon sink but led to SOC loss in deeper soil, as found through over 20 years of investigations in the United States (35). Therefore, current modes of cropland management worldwide cannot prevent warming-induced SOC losses in deeper soil. Given that SOC loss increases with soil depth, such loss in much deeper soils (1 to 3 m) would be significant. Together with greater SOC losses beneath the topsoil observed in England and Wales (6) and whole-soil-profile warming experiments in California forests (13), SOC changes may be more sensitive to warming in deeper soil than in well-studied topsoil worldwide (36). Especially, deeper SOC loss is extremely urgent in the black soil region of Northeast China (Fig. 3). Two reasons can explain such huge SOC loss in black soil despite that the Chinese Government attaches great importance to the protection of black land. First, black soil regions at high latitudes are exposed to higher rates of climate warming than other regions (*SI Appendix*, Fig. S2). Second, compared with other croplands, black soil has abundant SOC with shorter cultivation history (Fig. 1). Both climate warming and high original 1980 SOC stocks had a negative effect on SOC stock changes in this region (Fig. 2).

Meanwhile, we must acknowledge that our study has several limitations. This study has only two temporal SOC data points, although they were from a comparatively large-scale set of 205 sites across a variety of Chinese agroecosystem zones over the past four decades. In addition, although our resampling survey was based on study sites without transformation of land use and grain cropping systems, we could not explore the responses of the same cultivars or varieties because no historical records were available and current high-yield cultivars or varieties are in use. Obviously, different cultivars could alter their carbon allocation, leading to variations in both the quantity and quality of carbon inputs. Despite these limitations, our large-scale and one-of-a-kind resampling survey provides a unique reference for a better understanding of SOC dynamics in croplands globally.

Overall, preventing the loss of deeper SOC in croplands is a critical contribution to the 4 per mille initiative and China’s commitment to carbon neutrality, along with similar considerations for the topsoil carbon sink. Increasing organic matter inputs at deeper soil depths will thus be an effective climate solution pathway for cropland carbon sequestration (37, 38). For example, practices such as pulsing straw return, deep plowing to break up hardpans, and burying or embedding crop residues into the topsoil and subsoil have been shown to increase deeper SOC stocks despite priming effect-induced carbon decomposition (16). New deep-rooted crop varieties and trees or grasses in croplands can also enhance carbon inputs into deeper soil depths (37, 39). Furthermore, several other pathways have been proposed to mitigate climate change. Specifically, buried SOC would contribute to deeper soil carbon sinks (40). In addition, adding and mixing ameliorants, particularly the application of biochar (the carbonaceous residue of pyrolyzed organic materials under low-oxygen conditions), offer optimal mitigation potential (41). However, the biochar option has not yet been well demonstrated beyond research settings, given the cost–benefit trade-offs for its large-scale implementation (39).

## Materials and Methods

### Soil Resampling Campaigns.

We used data from the Second National Soil Survey of the Country, which was completed during 1979–1985. Soil sampling from croplands was conducted during 1979–1981, so we designated the year 1980 as the initial time point in this study. We utilized data from 1,023 soil profiles (sites) recorded in the survey (Fig. 1). Because of China’s rapid urbanization and population growth over the last four decades, we found that most of the original sites had been replaced by impermeable surfaces. Additionally, some sites had been converted to forests and grasslands due to ecological engineering programs such as the Grain for Green and Shelterbelt. Consequently, we were able to resample 205 sites between July and September 2023 that matched the original coordinates from 1980. We sampled soil from individual depths (0 to 20, 20 to 40, 40 to 60 cm, and 60 to 100 cm) within three 10 m × 10 m plots (replicates) established at each of the original sites. We centered one of the three sampling plots on the original coordinates from the 1980s and located the other two plots 10 m apart from this central plot. For each plot, we extracted seven soil samples and combined them to form a composite sample. Soil samples were air-dried, sieved (2 mm), and homogenized after the removal of fine roots and other debris. After removing inorganic carbon, we measured SOC using a Multi N/C 2100s analyzer (Analytik Jena AG, Germany). We calculated SOC stocks using the following equation (42):

[1] $${SOC}_{s}=SOC\times BD\times \left(1-f\right)\times T,$$

where SOC_s_ is SOC stocks and SOC is SOC concentration. BD, f, and T are soil bulk density, the volumetric percentage of coarse fraction, and the soil depth, respectively.

We further collected changes in the rate of SOC from published studies in Chinese and European farmland soils. Detailed data and information can be found in *SI Appendix*, Table S2.

Quantifying SOC stock changes using the fixed-depth approach was prone to errors, owing to changes in soil bulk density with shifts in SOC or management (43–45). The equivalent soil mass approach could estimate changes in SOC stocks relying on a consistent soil mass of fine earth (<2 mm), and this approach was not adversely affected by changes in bulk density (43, 45). However, taking the same sort of soil samples at different temporal data points was difficult, especially for a large-scale field study, which resulted in the fixed-depth approach being more widely used than the equivalent soil mass approach (45). We adopted the cumulative coordinates approach to overcome these problems and to facilitate the comparison of research conducted across different times, locations, soil conditions, and researchers (43). Following the cumulative coordinates approach, we first established the linear correlation between natural logarithm-transformed cumulative SOC stocks and natural logarithm-transformed cumulative fine earth mass along the soil profile in 2023 (43). We calculated the mass of fine earth as the difference between the bulk density and the mass of organic matter (the conversion factor from organic matter to SOC is 0.58). We then calculated SOC stocks with the same mass of fine earth in 1980 at soil depths of 0 to 20, 20 to 40, 40 to 60 cm, and 60 to 100 cm, respectively. We quantified changes in SOC stocks using the natural logarithm-transformed response ratio (lnRR):

[2] $$\text{ln}RR=\text{ln}\left(\frac{SOC\_2023}{SOC\_1980}\right),$$

where SOC_2023 and SOC_1980 are SOC stocks in 2023 and 1980, respectively.

### Model Selection Analysis.

To investigate the factors responsible for lnRR variations, we then considered MAT, MAP, changes in MAT and MAP between 1980 (over the last five years) and 2023 (over the last five years), and the extremely high-temperature and drought events during 1980–2023, management (straw return rate, fertilization, and grain yields), SOC_1980, net primary productivity, and soil properties (pH and texture). Historical annual temperatures and precipitation were obtained from WorldClim Version 2 (https://www.worldclim.com/). Historically monthly maximum temperatures of the warmest quarter were also obtained from the WorldClim Version 2. Following the definition of climate extremes from IPCC (46), the fraction of months (the warmest quarter) with maximum temperature above the 90th percentile with respect to the 1961–1980 reference period was defined as extremely high-temperature events. The standardized precipitation-evapotranspiration index (SPEI) provides a robust and accurate assessment of drought stresses across various regions, which can be applied for a range of applications including drought monitoring, risk assessment, and climate change studies (47). The fraction of SPEI being lower than two is defined as extreme drought events from 1980 to 2023 at a time scale of twelve months (48). The straw return data were available from the Ministry of Agriculture and Rural Affairs of China’s National Report on the Comprehensive Utilization of Crop Straw (http://zdscxx.moa.gov.cn:8080/nyb/pc/index.jsp). The grain yield, fertilization, and irrigation data were derived from the China Statistical Yearbooks of the National Bureau of Statistics of China (https://www.stats.gov.cn/sj/ndsj/). The averaged net primary productivity across 2001–2023 from a Moderate Resolution Imaging Spectroradiometer was also obtained.

To predict changes in SOC stocks, we also validated the performances of six typical machine learning algorithms, including 1) random forest, 2) extreme gradient boosting, 3) support vector machines, 4) recursive partitioning and regression trees, 5) neural networks, and 6) multivariable linear regression. Among them, the multivariable linear regression acquired the least Rms error (RMSE) across soil depths (*SI Appendix*, Table S3). The selection of the best multivariable linear model was then based on the Bayesian information criterion (BIC). The relative importance value for a particular predictor was equal to the sum of the BIC weights (probability that a model is the most plausible one) for the models in which the predictor appeared. Hence, a predictor that was included in models with large BIC weights received a high importance value. These values were regarded as the overall support for each variable across all models. Using this analysis, the best models (with lowest BIC) for predicting the lnRR variations were also obtained for soil depths of 0 to 20, 20 to 40, 40 to 60 cm, and 60 to 100 cm, respectively (*SI Appendix*, Table S1).

### Upscaling Model Predictions.

An optimal model from the model selection analysis was employed to estimate changes in SOC stocks at a national scale. First, the K-fold cross-validation (with K = 10) was used to validate the models (*SI Appendix*, Fig. S7). The slopes of the linear correlations between changes in the observed and predicted SOC stocks were consistently close to 1.00 for all four tested soil depths, indicating that the applied statistical model had no biases. Second, to generate spatially explicit and quantitative maps of changes in SOC stocks, the predictors were aggregated or resampled to a 0.01° × 0.01° resolution by soil depths (0 to 20, 20 to 40, 40 to 60, and 60 to 100 cm). The 100-time bootstrapping was applied to generate uncertainties. Specifically, for each bootstrap iteration, we first randomly sampled 90% of the data from the 205 sites, then established the best model for each soil depth using these sampled data, and finally predicted changes in SOC stocks for each bootstrap iteration.

## Supplementary Material

Appendix 01 (PDF)

Dataset S01 (CSV)

## Data Availability

All study data are included in the article and/or supporting information.
