# Supplementary material for: Carbon gain in upper but loss in deeper cropland soils across China over the last four decades
Source: Proc Natl Acad Sci U S A. 2024 Dec 31;122(1):e2422371122. doi: 10.1073/pnas.2422371122 (PMC11725835; doi:10.1073/pnas.2422371122)
Supplement: Supplementary file 1 — Appendix 01 (PDF) [file pnas.2422371122.sapp.pdf]

PNAS

Supporting Information for

Carbon gain in upper but loss in deeper cropland soils across China over the last four decades.

Zhenghu Zhou, Chuankuan Wang, Yue Li, Xuhui Wang, Xinhua He, Minggang Xu*, and Andong Cai*.

*Minggang Xu and Andong Cai.
Email: xuminggang@caas.cn and caiandong@caas.cn

This PDF file includes:

Tables S1-S3
Figures S1-S7

Other supporting materials for this manuscript include the following:

Dataset S1

Table S1. The best models for predicting changes in soil organic carbon (SOC) stocks.

Soil depths (cm)	Best models	BIC	R^2
0–20	lnRR ~ 0.90 – 0.80 SOC_1980 – 0.38 MAT + 0.77 NPP + 0.41 Straw	151.3	0.58
20–40	lnRR ~ 0.55 – 0.76 SOC_1980 – 0.42 MAT + 0.70 NPP + 0.25 Straw	120.7	0.59
40–60	lnRR ~ 0.50 – 0.75 SOC_1980 – 0.38 MAT + 0.61 NPP	168.1	0.56
60–100	lnRR ~ 1.49 – 0.75 SOC_1980 – 0.41 MAT – 0.23 ΔMAT	216.9	0.54

BIC, Bayesian information criterion; MAT, mean annual temperature; ΔMAT, changes in MAT in the past four decades; NPP, net primary productivity; SOC_1980, SOC stock in 1980 (natural logarithm-transformed); Straw, proportion of straw return.

Table S2. Soil organic carbon sequestration rates in croplands of China and the European countries.

Country	Methods	Period	Soil organic carbon sequestration rate (g m^{-2} yr^{-1})	Data sources
China	Survey	1991–2012	15.3	He et al., 2021(1)
China	Survey	1980–2008	7.4	Yan et al., 2011(2)
China	Survey	1980–2011	14.0	Zhao et al., 2018(3)
China	Meta-analysis	1993–2003	16.9	Huang and Sun, 2006(4)
China	Meta-analysis	1980–2006	19.2	Pan et al., 2010(5)
China	Meta-analysis	1980–2000	14.4	Sun et al., 2010(6)
China	Meta-analysis	1980–1990	18.6	Piao et al., 2009(7)
China	Meta-analysis	1980–2000	15.2	Xie et al., 2007(8)
China	Meta-analysis	1980–2000	8.4	Yu et al., 2009(9)
China	Model	–	–73.1	Li et al., 2003(10)
China	Model	–	25.9	She et al., 2017(11)
China	Model	–	–81.5	Tang et al., 2006(12)
China	Model	–	–154.0	Tang et al., 2010(13)
China	Model	–	18.3	Yu et al., 2012(14)
China	Model	–	11.3	Ren et al., 2011(15)
Belgium	Potential estimation	–	49.7	Rodrigues et al., 2021(16)
Denmark	Potential estimation	–	5.9	Rodrigues et al., 2021(16)
France	Potential estimation	–	20.0	Rodrigues et al., 2021(16)
France	Potential estimation	–	10.5	Rodrigues et al., 2021(16)
Germany	Potential estimation	–	10.6	Rodrigues et al., 2021(16)
Germany	Potential estimation	–	8.0	Rodrigues et al., 2021(16)
Ireland	Potential estimation	–	56.3	Rodrigues et al., 2021(16)
Ireland	Potential estimation	–	110.0	Rodrigues et al., 2021(16)
Italy	Potential estimation	–	0.1	Rodrigues et al., 2021(16)
Italy	Potential estimation	–	6.1	Rodrigues et al., 2021(16)
Italy	Potential estimation	–	31.1	Rodrigues et al., 2021(16)
Netherlands	Potential estimation	–	82.2	Rodrigues et al., 2021(16)
Netherlands	Potential estimation	v	10.4	Rodrigues et al., 2021(16)
Norway	Potential estimation	–	291.7	Rodrigues et al., 2021(16)
Norway	Potential estimation	–	3.3	Rodrigues et al., 2021(16)
Poland	Potential	–	15.4	Rodrigues et al.,

	estimation			2021(16)
Portugal	Potential estimation	–	177.8	Rodrigues et al., 2021(16)
Spain	Potential estimation	–	37.9	Rodrigues et al., 2021(16)
Sweden	Potential estimation	–	18.4	Rodrigues et al., 2021(16)
Sweden	Potential estimation	–	24.0	Rodrigues et al., 2021(16)
Switzerland	Potential estimation	v	210.0	Rodrigues et al., 2021(16)
Switzerland	Potential estimation	–	32.9	Rodrigues et al., 2021(16)

Data sources:

1. W. He*, et al.*, Soil organic carbon changes for croplands across China from 1991 to 2012. *Agronomy* **11**, 1433 (2021).
2. X. Yan, Z. Cai, S. Wang, P. Smith, Direct measurement of soil organic carbon content change in the croplands of China. *Global Change Biology* **17**, 1487-1496 (2011).
3. Y. Zhao*, et al.*, Economics- and policy-driven organic carbon input enhancement dominates soil organic carbon accumulation in Chinese croplands. *Proceedings of the National Academy of Sciences* **115**, 4045-4050 (2018).
4. Y. Huang, W. Sun, Changes in topsoil organic carbon of croplands in mainland China over the last two decades. *Chinese Science Bulletin* **51**, 1785-1803 (2006).
5. G. Pan, X. Xu, P. Smith, W. Pan, R. Lal, An increase in topsoil SOC stock of China's croplands between 1985 and 2006 revealed by soil monitoring. *Agriculture, Ecosystems & Environment* **136**, 133-138 (2010).
6. W. Sun, Y. Huang, W. Zhang, Y. Yu, Carbon sequestration and its potential in agricultural soils of China. *Global Biogeochemical Cycles* **24** (2010).
7. S. Piao*, et al.*, The carbon balance of terrestrial ecosystems in China. *Nature* **458**, 1009-1013 (2009).
8. Z. Xie*, et al.*, Soil organic carbon stocks in China and changes from 1980s to 2000s. *Global Change Biology* **13**, 1989-2007 (2007).
9. Y. Yu, Z. Guo, H. Wu, J. A. Kahmann, F. Oldfield, Spatial changes in soil organic carbon density and storage of cultivated soils in China from 1980 to 2000. *Global Biogeochemical Cycles* **23** (2009).
10. C. Li*, et al.*, Modeling soil organic carbon change in croplands of China. *Ecological Applications* **13**, 327-336 (2003).
11. W. She*, et al.*, Integrative analysis of carbon structure and carbon sink function for major crop production in China's typical agriculture regions. *Journal of Cleaner Production* **162**, 702-708 (2017).
12. H. Tang, J. Qiu, E. Van Ranst, C. Li, Estimations of soil organic carbon storage in cropland of China based on DNDC model. *Geoderma* **134**, 200-206 (2006).
13. H. Tang, J. Qiu, L. Wang, L. Hu, C. Li, E. Van Ranst, Modeling soil organic carbon storage and its dynamics in croplands of China. *Agricultural Sciences in China* **9**, 704-712 (2010).
14. Y. Yu, Y. Huang, W. Zhang, Modeling soil organic carbon change in croplands of China, 1980–2009. *Global and Planetary Change* **82**, 115-128 (2012).
15. W. Ren*, et al.*, Spatial and temporal patterns of CO2 and CH4 fluxes in China's croplands in response to multifactor environmental changes. *Tellus B: Chemical and Physical Meteorology* **63**, 222-240 (2011).
16. L. Rodrigues*, et al.*, Achievable agricultural soil carbon sequestration across Europe from country-specific estimates. *Global Change Biology* **27**, 6363-6380 (2021).

Table S3. The performance of six machine learning algorithms.

Models	0–20 cm	20–40 cm	40–60 cm	60–100 cm
Random forest	0.39	0.38	0.42	0.47
Extreme gradient boosting	0.38	0.40	0.41	0.47
Support vector machine	0.36	0.36	0.40	0.45
Recursive partitioning and regression tree	0.40	0.36	0.43	0.47
Neural network	0.35	0.34	0.36	0.44
Multivariable linear regression	0.34	0.32	0.36	0.40

The values are root mean square errors.

Figure S1. Changes in grain yields (A), fertilization (B), irrigation (C), and reservoir capacity (D) over the last four decades in China.

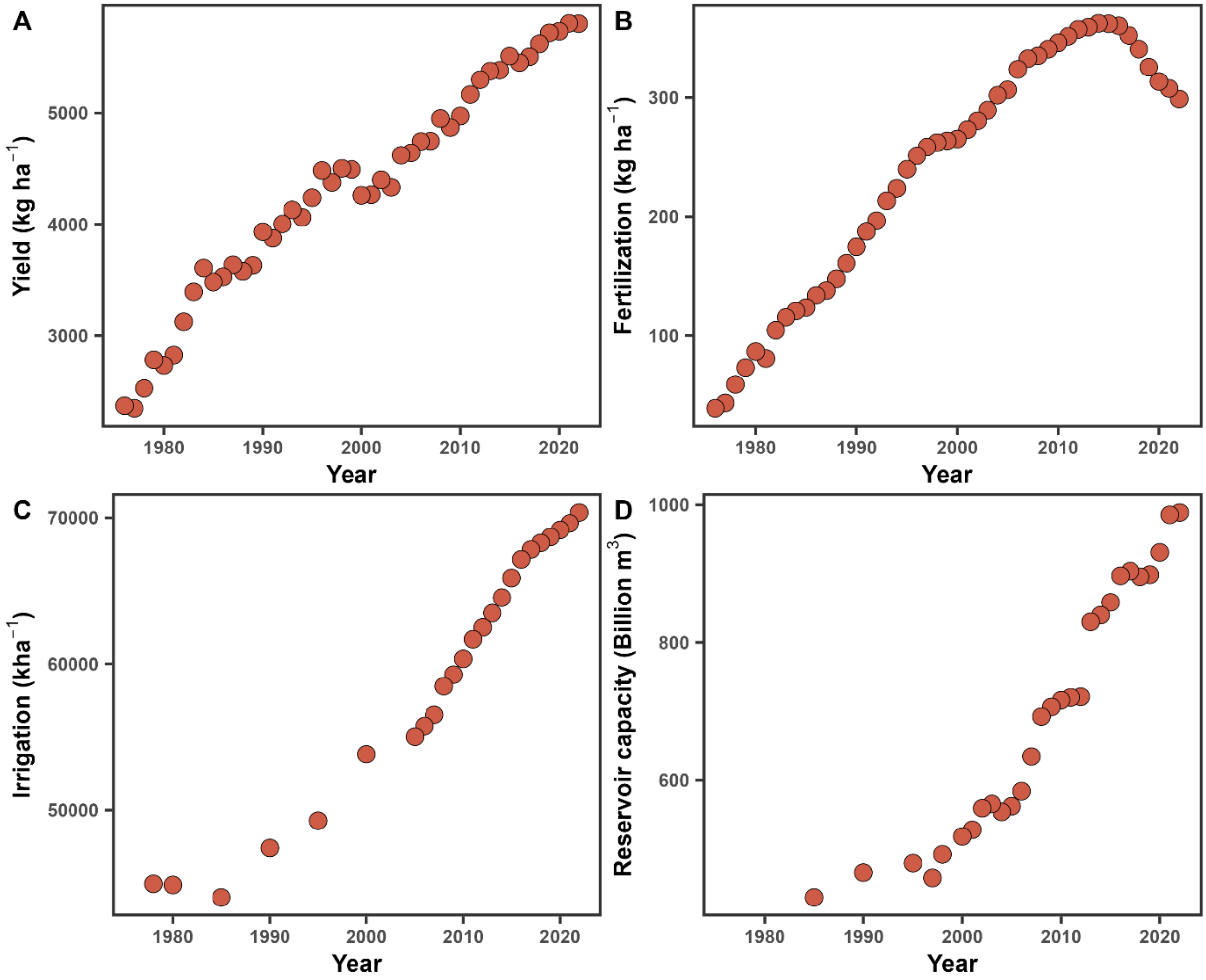

Figure S2. Climate change over the last four decades in China's croplands. (A) Mean annual temperature (MAT). (B) Changes in MAT (ΔMAT). (C) Mean annual precipitation (MAP). (D) Changes in MAP (ΔMAP). (E) Extreme high temperature events (ExtremeT). (F) Extreme drought events (ExtremeD).

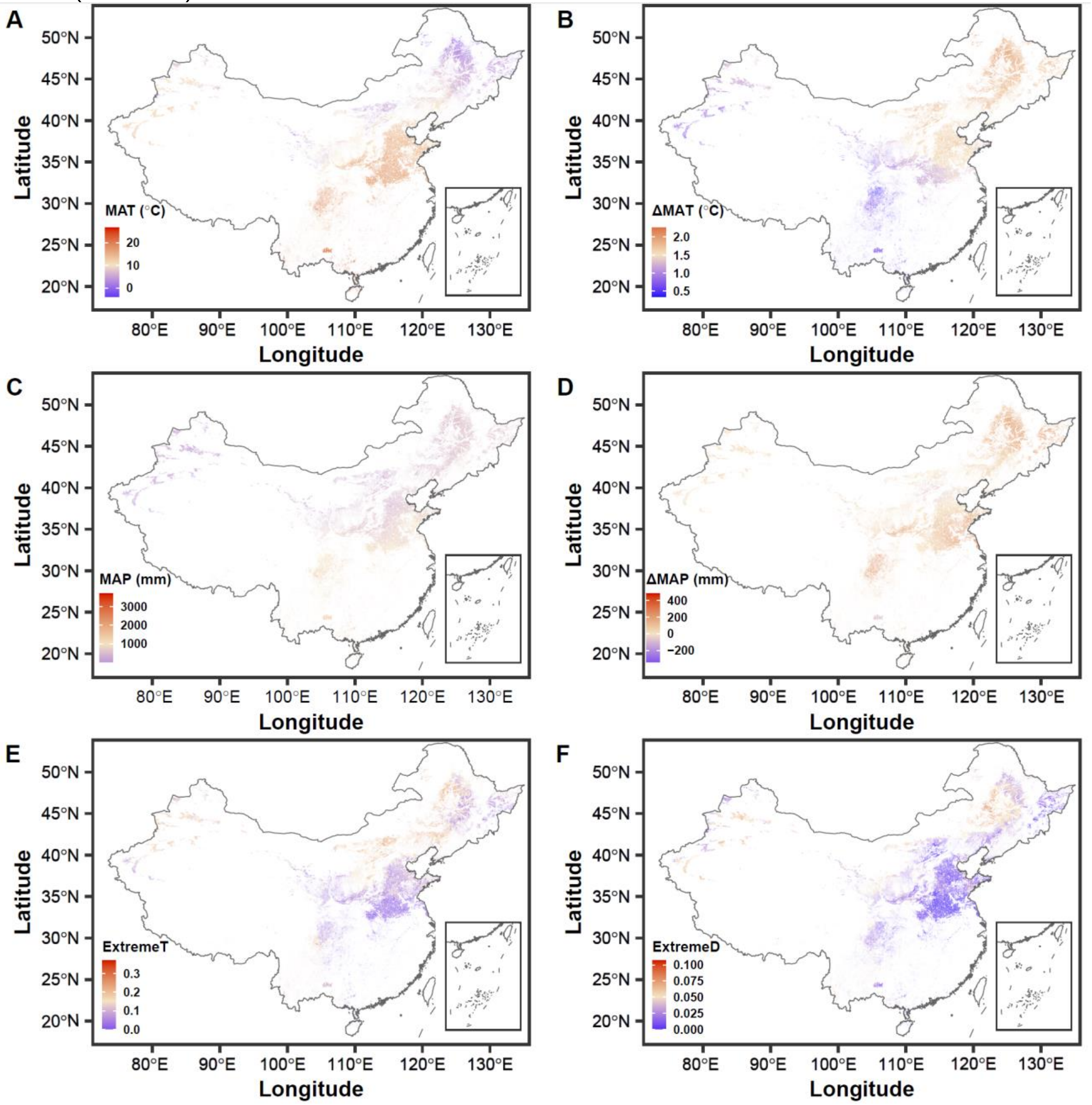

Figure S3. Changes in soil organic carbon (SOC) stocks for different soil depths. SOC_1980, SOC stocks in 1980. ΔSOC, changes in SOC over the last four decades. Panel (A), (B), (C), (D) and (E) respectively show ΔSOC at 0–20 cm, in 20–40 cm, 40–60 cm, 60–100 cm, and *t*-test of ΔSOC for different soil depths. Encircled asterisks indicate significant changes (*P* < 0.05, *t*-test).

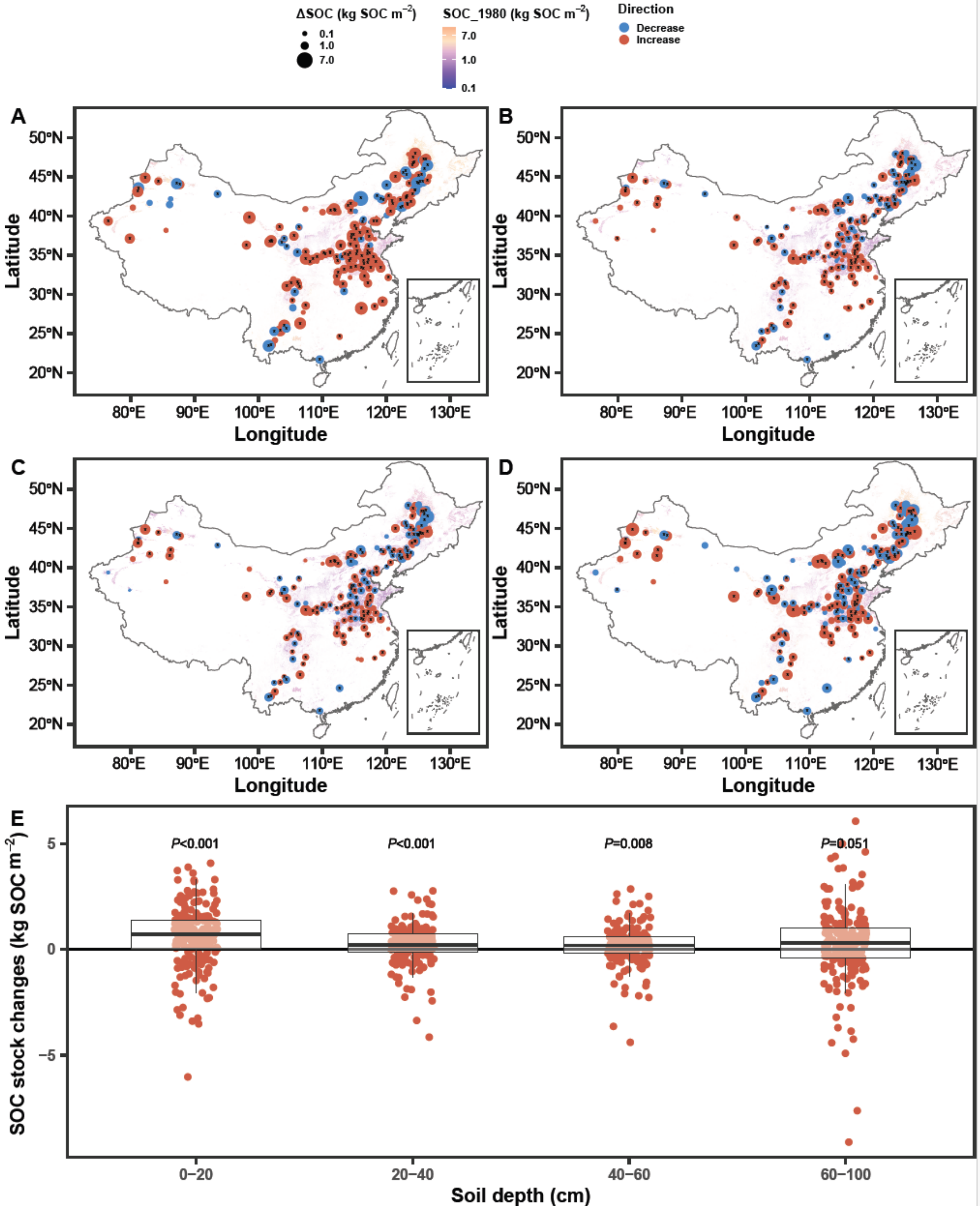

Figure S4. Contributors to changes in soil organic carbon (SOC) for soil depths of 0–20 (A), 20–40 (B), 40–60 (C), and 60–100 cm (D). Model-averaged importance of predictors for changes in SOC stocks based on the sum of Bayesian weights derived from model selection using Bayesian information criterion. SOC_1980, SOC stocks in 1980. NPP, net primary productivity. MAT, mean annul temperature. MAP, mean annul precipitation. ΔMAT, changes in MAT over the last four decades. ΔMAP, changes in MAP over the last four decades. ΔFertilization, changes in fertilization over the last four decades. ΔYield, changes in grain yields over the last four decades. Straw, straw return proportions. N = 205.

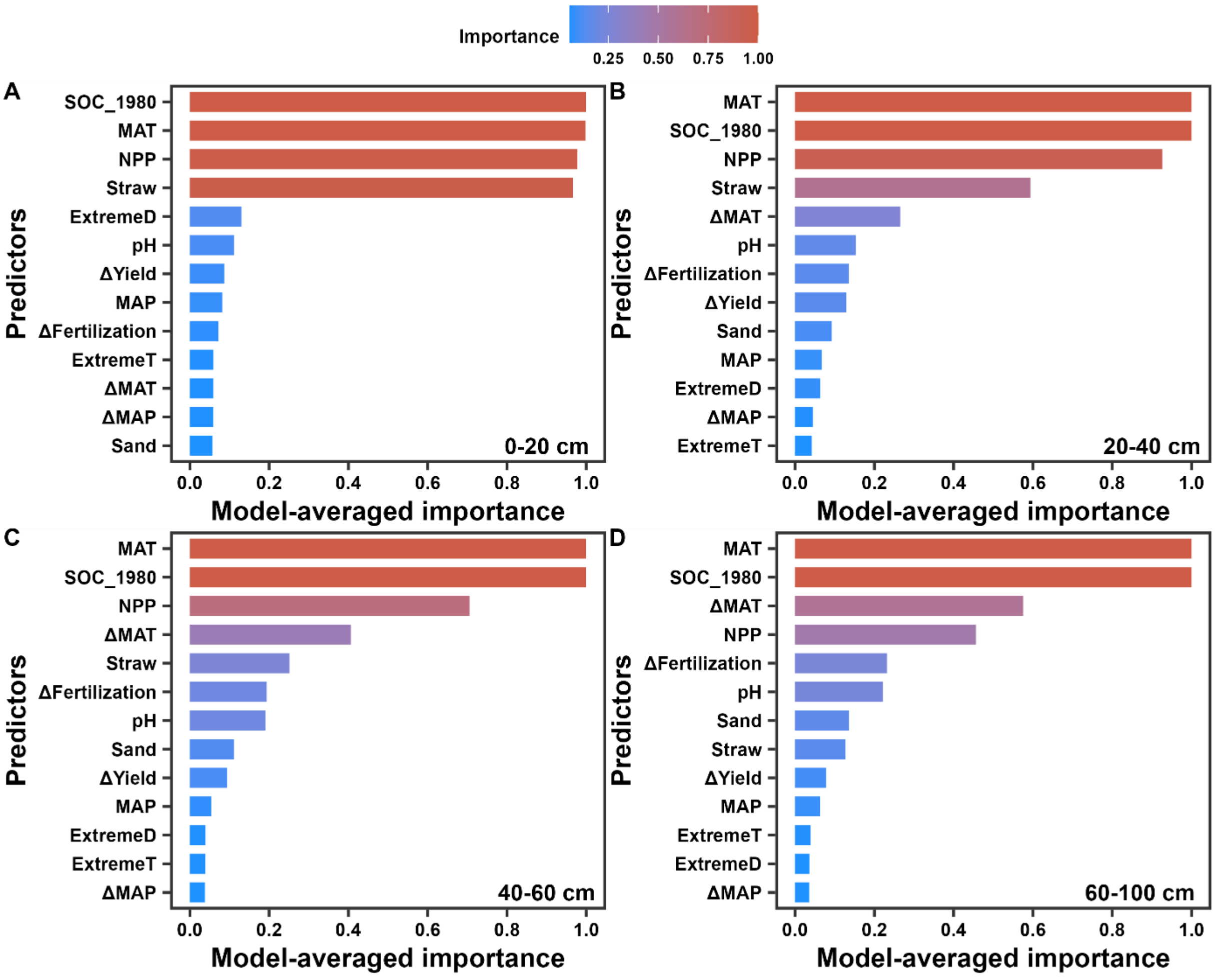

Figure S5 Effects of original soil organic carbon (SOC) on changes in soil organic carbon stocks for soil depths of 0–20 (A), 20–40 (B), 40–60 (C), and 60–100 cm (D). SOC_1980, original SOC stocks in 1980. lnRR, natural logarithm-transformed response ratios of SOC stocks in 2023 to SOC stocks in 1980. N = 205.

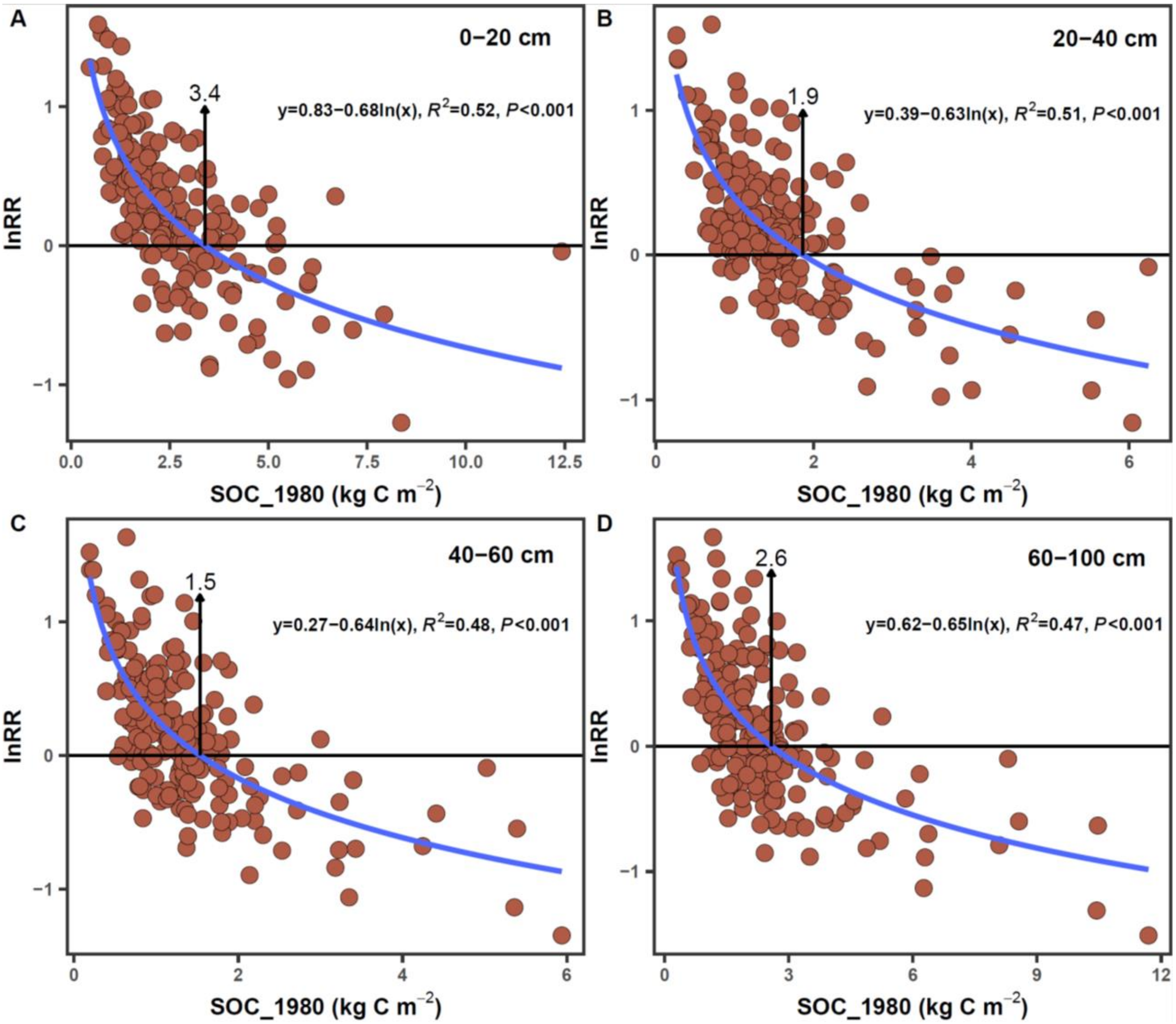

Figure S6. Effects of extreme high temperature (A) and drought (B) events on changes in soil organic carbon stocks. lnRR, natural logarithm-transformed response ratio of soil organic carbon stocks in 2023 to soil organic carbon stocks in 1980.

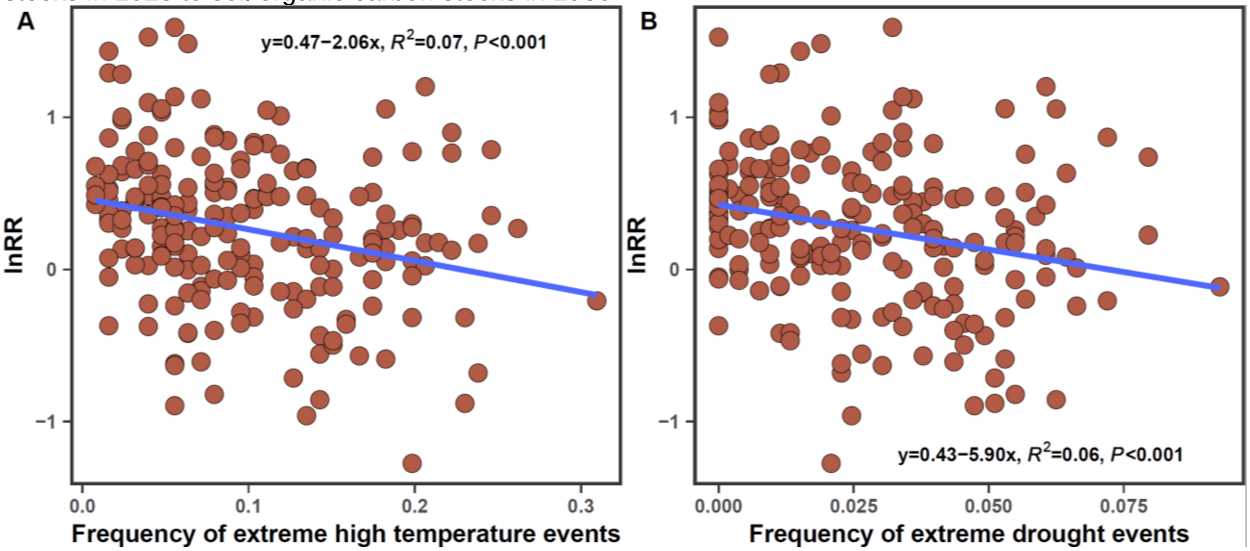

Figure S7. The 10-fold cross-validation of the best model for soil depths of 0–20 (A), 20–40 (B), 40–60 (C), and 60–100 (D) cm. lnRR, natural logarithm-transformed response ratio of soil organic carbon stocks in 2023 to soil organic carbon stocks in 1980.

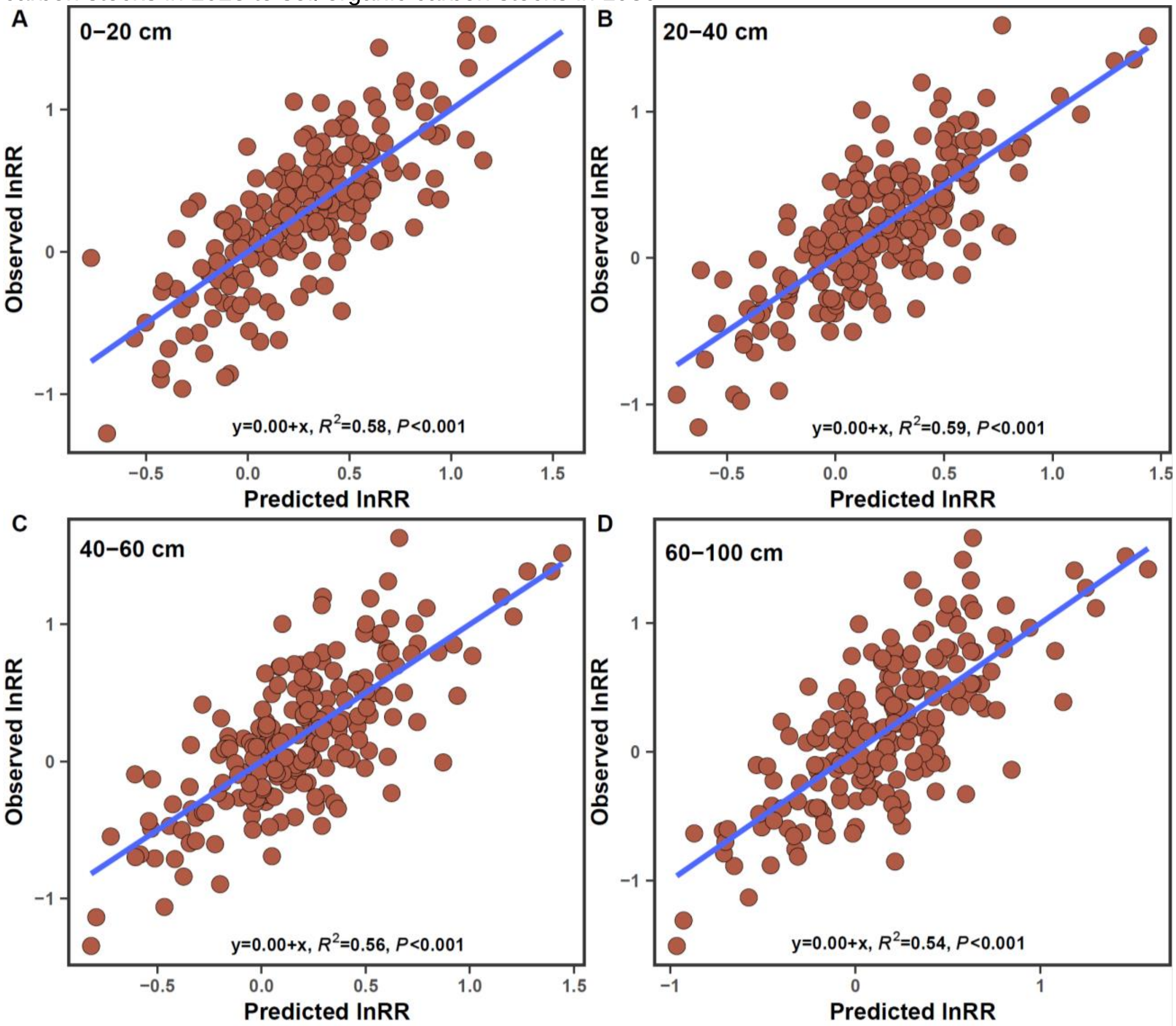

Dataset S1. All dataset supported for current study.
